# Indirect bilirubin mitigates tubular vacuolization and injury in experimental contrast nephropathy

**DOI:** 10.1186/s12882-026-05250-z

**Published:** 2026-08-05

**Authors:** Fatih Ünal, Mehmet Timur Selçuk, Samet Ece, Şefika Karabulut, Candan Altuntaş, Sevil Atalay Vural

**Affiliations:** 1 Department of Cardiology, The University Medical Centre Mannheim, Mannheim, Germany; 2 Department of Cardiology, Ankara City Hospital, Ankara, Türkiye; 3 Northbiome Ar-ge, Ankara, Türkiye; 4 Faculty of Pharmacy, Department of Pharmaceutical Basic Sciences, Gazi University, Ankara, Türkiye; 5 Center for Stem Cell and Gene Therapies Research and Practice, Kocaeli University, İzmit, Kocaeli Türkiye; 6 Veterinary Faculty, Department of Pathology, Ankara University, Ankara, Türkiye

**Keywords:** Contrast-associated acute kidney injury, Contrast media, Indirect bilirubin, Renal tubular injury, Histopathology, Oxidative stress, Apoptosis, Inflammation, Experimental nephropathy

## Abstract

Contrast-associated acute kidney injury (CA-AKI) remains a clinically relevant complication of intravascular contrast media use, involving complex and incompletely understood mechanisms including oxidative stress, inflammation, apoptosis, and vasomotor dysregulation. Indirect bilirubin has been proposed as a potential cytoprotective molecule due to its antioxidant and anti-inflammatory properties. In this experimental study, twenty-four male Wistar albino rats were randomized into three groups: sham, contrast nephropathy (CN), and contrast nephropathy treated with indirect bilirubin (CNIB). CA-AKI was induced using indomethacin, Nω-Nitro-L-Arginin-Methylester (L-NAME), and iodinated contrast media. Indirect bilirubin (30 mg/kg, intraperitoneal [i.p.]) was administered after injury induction. Renal function was assessed using serum creatinine (SCR), blood urea nitrogen (BUN), and urinary parameters. Histopathological injury was evaluated using semi-quantitative tubular damage scoring, and molecular analyses included inflammatory, apoptotic, and anti-apoptotic markers. Contrast media administration induced significant impairment in renal function and marked tubular injury compared with sham animals. Indirect bilirubin administration was associated with a reduction in histological tubular damage and vacuolization compared to untreated CA-AKI animals. However, improvements in Scr, BUN, and urinary indices did not reach statistical significance. In addition, expression patterns of inflammatory and apoptotic markers were inconsistent between functional, histological, and molecular outcomes, and did not uniformly support a classical anti-inflammatory or anti-apoptotic mechanism. Indirect bilirubin was associated with attenuation of histological renal injury in an experimental model of contrast-associated acute kidney injury; however, this effect was not accompanied by consistent functional improvement or coherent modulation of inflammatory and apoptotic pathways. These findings suggest a potential structural protective signal of indirect bilirubin in renal tissue, while highlighting the need for further studies to clarify underlying mechanisms and functional relevance.

## Summary at a glance

Contrast-induced nephropathy is a clinically relevant complication of contrast media that can lead to acute kidney failure and the need for hemodialysis, particularly in radiological imaging and invasive cardiology.

In this experimental rat study, contrast-induced nephropathy was established using pharmacological inhibition of renal prostaglandins and nitric oxide, followed by contrast media administration.

Contrast media exposure induced intracellular vacuolization and tubular cellular damage in renal tissue and resulted in impaired renal function.

Indirect bilirubin treatment significantly reduced tubular damage and renal vacuolization and improved renal function.

These findings suggest that indirect bilirubin may exert renoprotective effects in contrast-induced nephropathy; however, the underlying molecular mechanisms remain unclear and warrant further investigation.

## Introduction

Contrast-associated acute kidney injury (CA-AKI) is a clinically important complication that may occur following intravascular administration of iodinated contrast media during diagnostic and interventional radiological procedures, including computed tomography and percutaneous coronary interventions. Although its incidence varies depending on patient risk profile, CA-AKI remains a relevant cause of hospital-acquired renal dysfunction, particularly in individuals with pre-existing chronic kidney disease, diabetes mellitus (DM), heart failure, or exposure to nephrotoxic agents [1–5]. Contrast Media (CM) is the third most common cause of acute renal failure after ischemic nephropathy and toxic nephropathy [2].

The pathogenesis of CA-AKI is multifactorial and complex, involving mechanisms such as apoptosis, necrosis, oxidative stress, vasculopathy and vasoconstriction, pro-inflammatory responses, shear stress due to the high viscosity of contrast agents, intracellular danger signaling pathways, hypoxia, and hyperosmolarity [6–8]. These processes may interact in a patient-specific manner, influenced by baseline renal function and comorbid conditions such as DM, atherosclerosis, exposure to nephrotoxic drugs and hypertension; resulting in heterogeneous clinical presentations [3]. Despite extensive preclinical and clinical research, effective pharmacological strategies for the prevention of CA-AKI remain limited. Intravenous hydration remains the most consistently supported preventive measure, while other approaches such as statins, N-acetylcysteine, and vasodilatory agents have shown variable or inconsistent efficacy across studies [1, 9–11]. Consequently, there is ongoing interest in identifying novel agents that may mitigate renal injury associated with contrast exposure.

Indirect bilirubin (IB) is a hydrophobic molecule generated during the catabolism of heme via the enzymatic actions of heme oxygenase and biliverdin reductase [12]. While elevated concentrations of IB are cytotoxic, lower concentrations exhibit cytoprotective properties [13]. These protective effects are predominantly mediated through its antioxidant activity - by scavenging and reducing reactive oxygen species (ROS) such as superoxide and peroxynitrite - as well as its anti-inflammatory and anti-apoptotic actions, which involve the suppression of pro-inflammatory, apoptotic, and intracellular danger signaling pathways (e.g., Nuclear factor kappa-light-chain-enhancer of activated B cells [NF-κB], caspases, tumor necrosis factor-alpha [TNF-alpha]) [14–19]. Therefore, the present study aimed to investigate the potential association between IB administration and renal structural and functional changes in an experimental model of contrast-associated acute kidney injury in rats, with a focus on histopathological injury, renal function parameters, and selected inflammatory and apoptotic markers.

## Results

### Follow up rat characteristics

One sudden death was observed in each of the contrast nephropathy (CN) and contrast nephropathy treated with indirect bilirubin (CNIB) groups following i.p. administration of L-NAME and indomethacin. Additionally, two more rats - one from the CN group and one from the CNIB group - died after intravenous iohexol administration via the tail vein. Necropsy examination was performed in all four rats (Table 1).

In one rat from the CN group that died after L-NAME and indomethacin administration, tracheal and lingual edema was observed. In the remaining three rats, diffuse macroscopic necrotic changes were detected in the liver, lungs, and kidneys.

**Table 1:** Survival status of experimental groups

Group	Initial *n*	Survived *n*	Mortality *n*	Survival rate %
Sham	8	8	0	100
CN	8	6	2	75
CNIB	8	6	2	75

CN: contrast nephropathy; CNIB: contrast nephropathy + indirect bilirubin treatment

### Renal function parameters, water intake and urine output

**Fig. 1:**
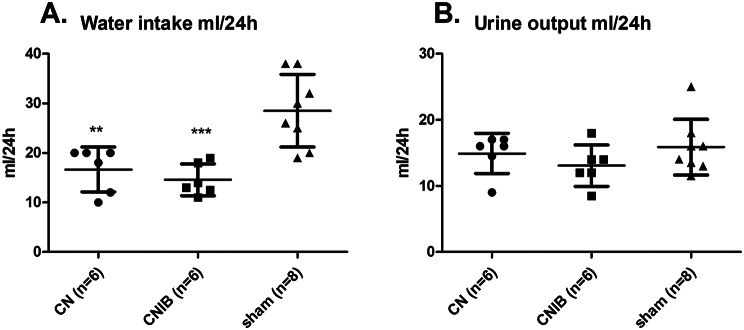
Metabolic cage, water intake and urine output in 24 hours: Panel **A** shows 24-hour water intake, and Panel **B** shows 24-hour urine output. The distribution of water intake data was parametric, whereas urine output data followed a non-parametric distribution. Accordingly, group comparisons and post-hoc analysis for water intake were performed using one-way ANOVA followed by the LSD test, while urine output was analyzed using the Kruskal-Wallis test followed by Bonferroni-corrected Mann-Whitney U tests. **A**: Group analysis using one-way ANOVA revealed a significant difference (*p* < 0.001, ***); post-hoc analysis with LSD showed significant differences between the CN and sham groups (*p* = 0.001, **) and between the CNIB and sham groups (*p* < 0.001, ***), while there was no significant difference between the CN and CNIB groups (*p* = 0.526). **B**: Group analysis using the Kruskal-Wallis test was not significant (*p* = 0.377); post-hoc analysis with Mann-Whitney U tests showed no significant differences between CN and sham (*p* = 0.794), CNIB and sham (*p* = 0.269), or CN and CNIB (*p* = 0.197). CN: contrast-induced nephropathy; CNIB: contrast-induced nephropathy + indirect bilirubin (30 mg/kg, i.p.). Bars represent mean ± SEM for water intake (**A**) and median with min - max values for urine output (**B**)

**Fig. 2:**
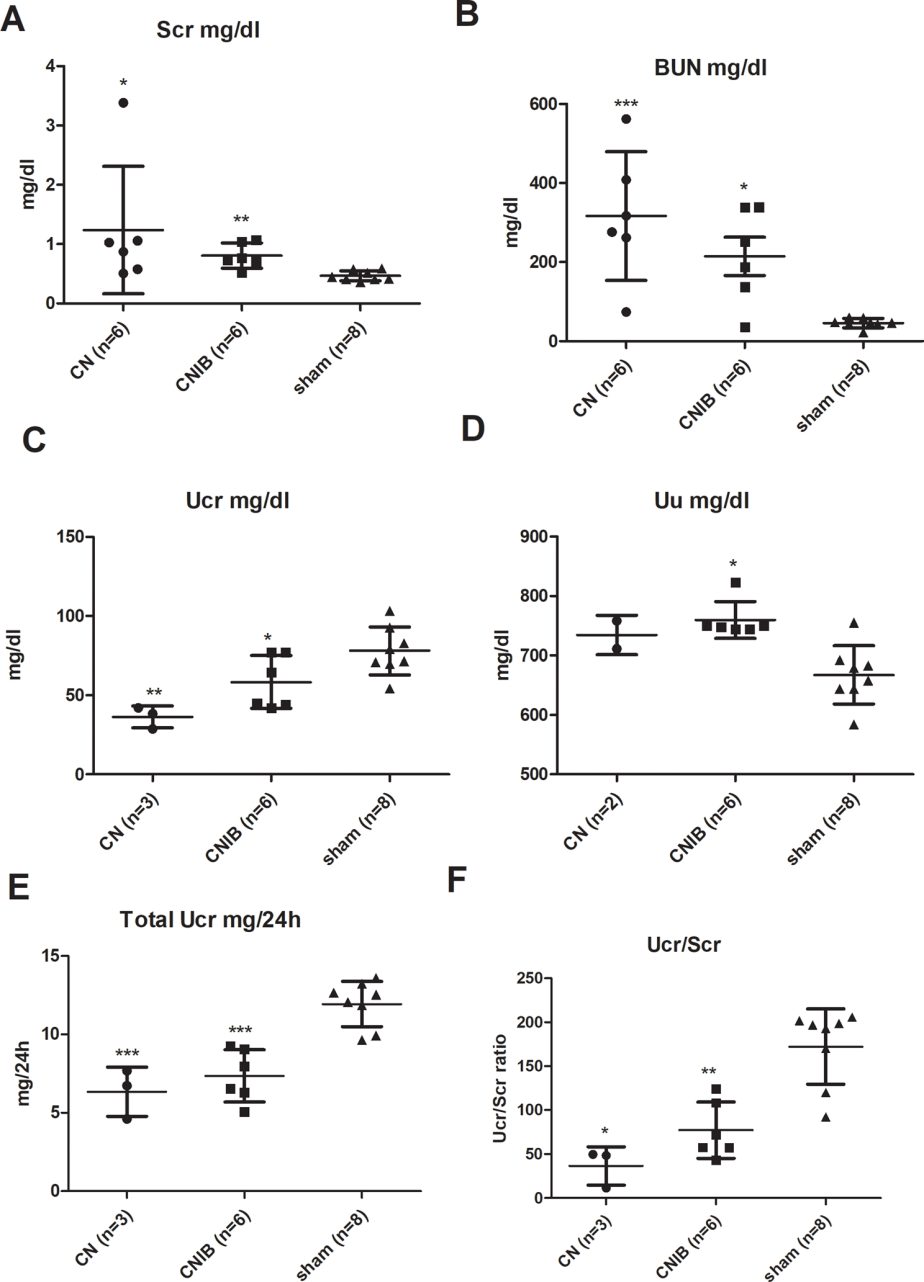
Serum and urine metabolites and renal function parameters. Scr (**A**): serum creatinine; BUN (**B**): blood urea nitrogen; Ucr (**C**): urine creatinine; Uu (**D**): urine urea; total Ucr (**E**): total urine creatinine; Ucr/Scr (**F**): urine creatinine/serum creatinine ratio. CN: contrast-induced nephropathy; CNIB: contrast-induced nephropathy treated with indirect bilirubin (30 mg/kg, i.p.). Parametric data are presented as mean ± SD, and non-parametric data as median (min–max). *p < 0.05, **p < 0.01, ***p < 0.001 versus sham group

To assess hydration status and renal excretory function, 24-hour water intake and urine output were evaluated. The 24-hour water intake was significantly reduced in both the CN and CNIB groups compared to the sham group (*p* < 0.05). However, there was no significant difference between the CN and CNIB groups (Fig. 1A). In contrast, the group analysis for 24-hour urine output did not show a statistically significant difference among the groups (Fig. 1B).

To evaluate renal functional impairment induced by contrast exposure, serum and urinary biochemical parameters were analyzed. Scr and BUN levels were significantly increased in both the CN and CNIB groups compared to the sham group (*p* < 0.05) (Fig. 2A and B), whereas urinary creatinine (Ucr) (Fig. 2C), 24-hour urinary creatinine excretion (24 h Ucr) (Fig. 2E), and the Ucr/Scr ratio (Fig. 2F) were significantly decreased (*p* < 0.05).

To evaluate renal functional impairment induced by contrast exposure, serum and urinary biochemical parameters were analyzed. Scr and BUN levels were significantly increased in both the CN and CNIB groups compared to the sham group (*p* < 0.05) (Fig. 2A and B). In contrast, urinary creatinine (Ucr), 24-hour urinary creatinine excretion (24 h Ucr), and the Ucr/Scr ratio were significantly decreased in both contrast-exposed groups compared to the sham group (*p* < 0.05) (Fig. 2C and E, and 2F).

These findings indicate impaired renal excretory function following contrast media administration.

To further evaluate urinary metabolic alterations associated with renal injury, urinary urea (Uu) levels were analyzed. There was no statistically significant difference in Uu levels between the CN group and either the sham group or the CNIB group. However, Uu levels were significantly higher in the CNIB group compared to the sham group (*p* < 0.05) (Fig. 2D).

To evaluate the potential effects of indirect bilirubin treatment on renal functional parameters, biochemical findings of the CNIB group were compared with those of the untreated CN group. Treatment with indirect bilirubin (30 mg/kg, i.p.) partially attenuated contrast-induced renal dysfunction by increasing urinary creatinine (Fig. 2C), 24-hour urinary creatinine excretion (Fig. 2E), and the Ucr/Scr ratio (Fig. 2F), while reducing Scr (Fig. 2A) and BUN (Fig. 2B) levels compared to the untreated CN group. Nevertheless, these changes did not reach statistical significance.

### Histopathological and immunohistological changes in renal tissue

**Fig. 3:**
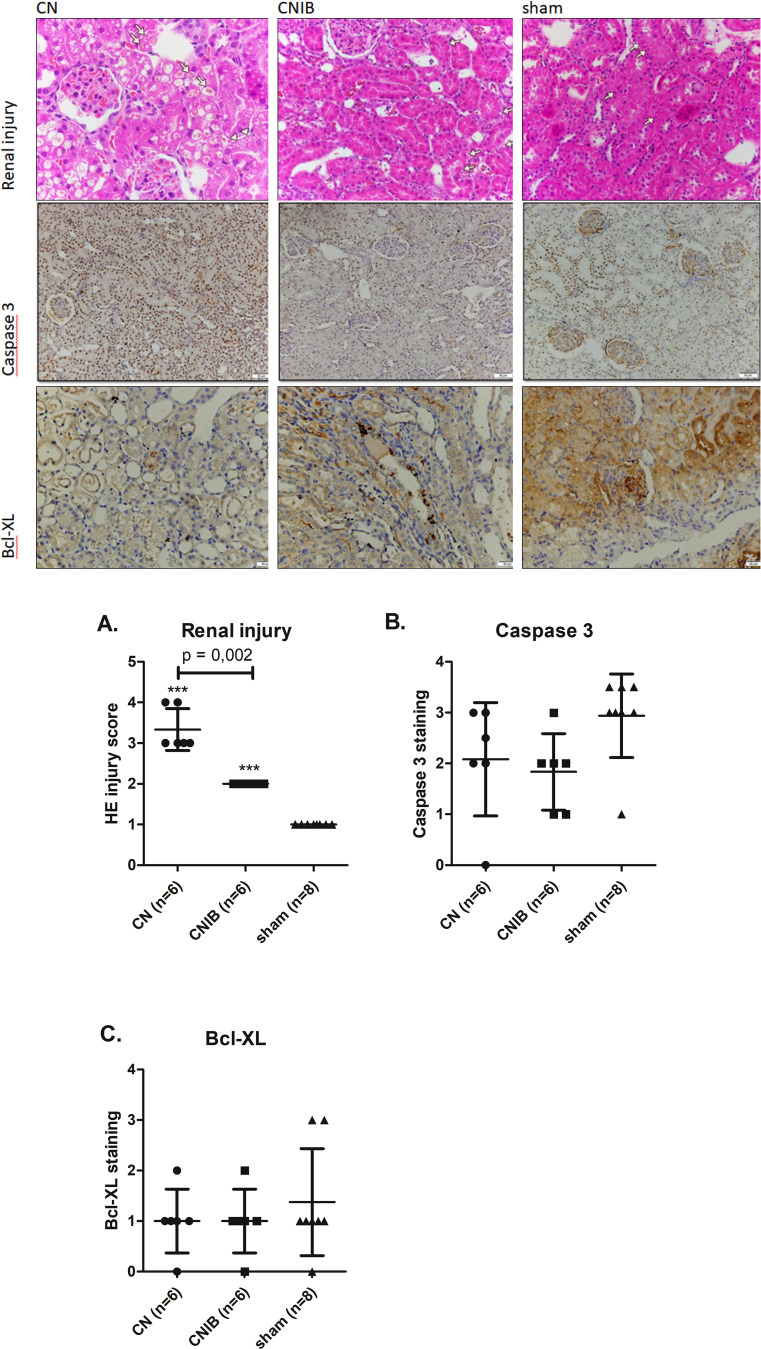
Histopathological changes and immunohistochemical staining of caspase-3 and Bcl-XL in renal tissue: Renal injury and immunohistochemical changes were evaluated using HE, caspase-3, and Bcl-XL staining of kidney tissue sections. According to the Shapiro-Wilk normality test; HE injury score, caspase-3 and Bcl-Xl values were non-parametric. **A**: group analysis with Kruskal-Wallis (*p*<0.001, ***); post-Hoc analysis with Mann Whitney U corrected with Banferroni CN versus sham (*p*<0.001, ***), CNIB versus sham (*p*<0.001, ***), CN versus CNIB (*p*=0.002, **). **B**: group analysis with Kruskal-Walis (*p*=0.032, *); post-Hoc analysis with Mann Whitney U corrected with Bonferroni CN versus sham (p=0.043), CNIB versus sham (*p*=0.023), CN versus CNIB (*p*=0.402). **C**: group analysis with Kruskal-Wallis (*p*=0.817). All values are presented as median (min - max). CN, contrast nephropathy; CNIB, contrast nephropathy + indirect bilirubin (30 mg/kg IP). HE renal injury (**A**); caspase-3 staining (**B**); Bcl-XL staining (**C**)

To evaluate structural renal injury induced by contrast exposure and the potential histopathological effects of indirect bilirubin treatment, renal tissue sections were examined semi-quantitatively. IB treatment (30 mg/kg) significantly reduced tubular damage and vacuolization in renal tissue compared to the untreated CN group. Both the treated (CNIB) and untreated (CN) contrast nephropathy groups showed significantly greater tubular injury and vacuolization compared to the sham group (Fig. 3A).

To investigate apoptotic and anti-apoptotic changes associated with contrast-induced renal injury, immunohistochemical staining for caspase-3 and Bcl-XL was performed. Immunohistochemical analysis showed that caspase-3 and Bcl-XL staining in the sham group was trend towards increase but did not reach statistical significance (Fig. 3B and C).

To further evaluate apoptotic and anti-apoptotic responses in renal tissue, caspase-3 and Bcl-XL immunostaining analyses were performed. Although overall group comparison for caspase-3 staining revealed a statistically significant difference, post-hoc pairwise comparisons did not demonstrate significant differences between individual groups (Fig. 3B). No significant difference was observed among the groups for Bcl-XL staining (Fig. 3C).

### Gene expression of cytochrome C, TNF-α, IL-1β and IL-6

**Fig. 4:**
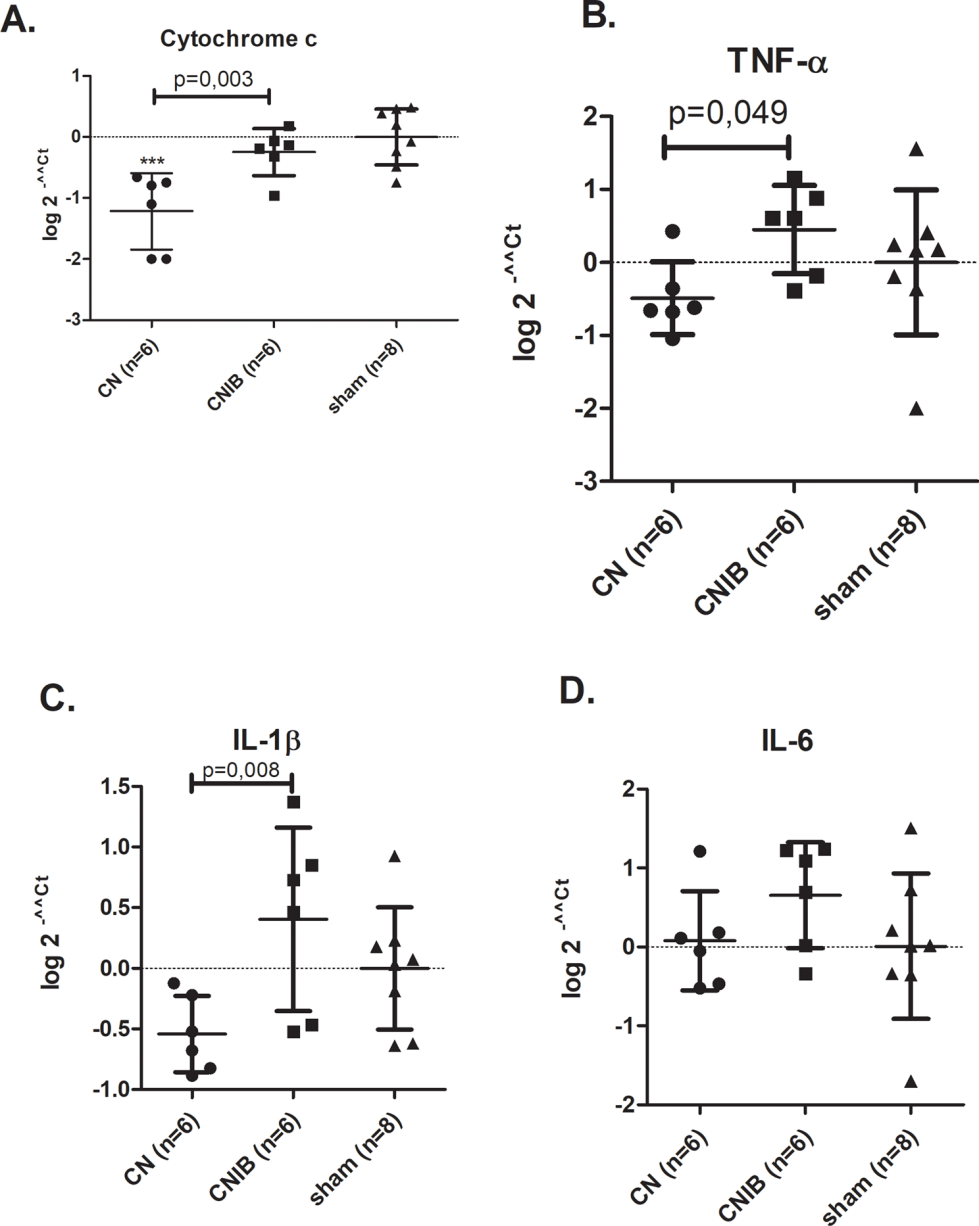
Gene expression of Cytochrome C, TNF-α, IL-1β and IL-6 in renal tissue: Gene expression analysis was performed using the qPCR method. The expression levels of Cyt-c, TNF-α, IL-1β, and IL-6 were calculated using the log 2-ΔΔCT method. The Shapiro-Wilk test showed that all gene expression values were normally distributed. Therefore, parametric tests were used for analysis. **A**: For Cyt-c expression, one-way ANOVA showed a significant difference between the groups (*p* = 0.001, **) Post-hoc analysis with LSD showed that the CN group had significantly lower expression than the sham group (*p* < 0.001, **) There was no significant difference between the CNIB and sham groups (*p* = 0.371). The CN group also had significantly lower expression than the CNIB group (*p* = 0.003, **) **B**: For TNF-α expression, one-way ANOVA showed no significant difference between the groups (*p* = 0.136). Post-hoc analysis showed no significant differences between CN and sham (*p* = 0.254), CNIB and sham (*p* = 0.294). CN had significantly lower TNF-α expression than CNIB (*p* = 0.049, **) **C**: For IL-1β expression, one-way ANOVA showed a significant difference between the groups (*p* = 0.027, **) Post-hoc analysis showed no significant difference between CN and sham (*p* = 0.085), or CNIB and sham (*p* = 0.190). However, the CN group had significantly lower expression than the CNIB group (*p* = 0.008, **) **D**: For IL-6 expression, one-way ANOVA showed no significant difference between the groups (*p* = 0.286). The results are presented as mean ± standard deviation. CN refers to contrast-induced nephropathy. CNIB refers to contrast-induced nephropathy treated with indirect bilirubin (30 mg/kg, i.p.)

To investigate molecular inflammatory and apoptotic responses associated with contrast-induced renal injury and indirect bilirubin treatment, renal gene expression analyses were performed. Group analysis revealed that the expression levels of Cyt-c (Fig. 4A) and IL-1β (Fig. 4C) were significantly different among the groups (*p* < 0.05). In contrast, the group differences for TNF-α and IL-6 (Fig. 4B and D) expression were not statistically significant. Interestingly, the CNIB group showed significantly higher gene expression levels of Cyt-c, TNF-α, and IL-1β compared to the untreated CN group (*p* < 0.05) (Fig. 4A, B and C). No statistically significant differences were found between the sham group and the CNIB group in terms of Cyt-c, TNF-α, or IL-1β expression (Fig. 4A, B and C). Neither the overall group comparison nor the post-hoc analysis for IL-6 expression revealed any significant differences (Fig. 4D). Both the sham group (which did not undergo the CN protocol) and the CNIB group exhibited significantly higher Cyt-c and IL-1β expression levels compared to the CN group (*p* < 0.05) (Fig. 4A and C).

## Discussion

CA-AKI is a clinically important complication of iodinated CM administration and remains a relevant cause of iatrogenic renal dysfunction in both diagnostic and interventional radiology as well as invasive cardiology. Despite its widespread use, CM exposure is associated with an increased risk of acute kidney injury, particularly in patients with predisposing conditions such as DM, chronic kidney disease, congestive heart failure, and concomitant exposure to nephrotoxic agents^6^,^20^. The pathogenesis of CA-AKI is complex and multifactorial, involving renal vasoconstriction, medullary hypoxia, oxidative stress, inflammatory activation, and direct tubular epithelial injury, ultimately leading to both structural and functional impairment of renal tissue. Although several preventive strategies have been proposed, including intravenous hydration, statin therapy, N-acetylcysteine, and vasodilator agents, their efficacy remains partial and no specific pharmacological therapy has been established to reliably prevent CA-AKI^2^,^4^,^11^,^21^. In this context, endogenous cytoprotective molecules have gained increasing attention. IB, a heme degradation product, has been reported to exert anti-inflammatory, antioxidant, and anti-apoptotic effects in various experimental models, suggesting a potential renoprotective role under conditions of oxidative and ischemic stress such as contrast exposure [13–16, 19].

Previous studies have demonstrated a protective effect of IB in various experimental models, including diabetic nephropathy [22,23], ischemia-reperfusion renal injury [16], adenine-induced nephropathy [24], and cisplatin nephrotoxicity [25]. In a clinical study, Huang et al. reported a negative association between serum IB levels and the incidence of contrast-induced nephropathy in patients undergoing coronary interventions [26]. In addition, Gao et al. showed that hemin, an inducer of heme oxygenase-1 - which catalyzes the conversion of heme into biliverdin and subsequently bilirubin - significantly attenuated contrast-induced nephropathy (CIN), also referred to as contrast-associated acute kidney injury (CA-AKI), in experimental models [27].

In the present study, the CN protocol successfully induced renal injury, as demonstrated by increased Scr and BUN levels together with decreased Ucr, Uu, total 24-hour urinary creatinine excretion, and Ucr/Scr ratio compared with the sham group. These findings are consistent with impaired renal function and support the validity of the experimental CA-AKI model.

Following administration of IB (30 mg/kg, i.p.), Scr and BUN levels showed a decreasing trend, whereas Ucr, 24-hour urinary creatinine excretion and the Ucr/Scr ratio tended to increase compared with untreated CN animals. Although these changes did not reach statistical significance, they may suggest a partial functional protective effect of IB on renal injury. The selected dose was based on the preclinical study by Vogel et al [28].

Urinary urea levels were similar between the treated and untreated groups. To the best of our knowledge, no previous in vivo experimental study has directly evaluated the effects of exogenous IB administration in a CA-AKI model. Therefore, this study may provide the first in vivo preclinical evidence suggesting a direct effect of administered IB on CA-AKI. In a related study, Gao et al. demonstrated that increased endogenous IB levels, induced via hemin-mediated activation of heme oxygenase-1 (HO-1), improved renal function in diabetic rats with CA-AKI by reducing Scr levels and enhancing creatinine clearance [27]. In addition, previous experimental studies have reported renoprotective effects of IB in models of adenine-induced nephrotoxicity [24], cisplatin-induced nephrotoxicity [25], and diabetic nephropathy [22], including improvements in Scr and urea levels.

Histopathological evaluation using H&E staining and a semi-quantitative scoring system demonstrated that IB treatment significantly reduced tubular damage and cellular vacuolization in the CN group compared with the untreated CN group. The sham group exhibited minimal tubular injury and significantly lower vacuolization compared with both CN groups.

Previous studies have demonstrated that the pathophysiology of contrast-associated acute kidney injury (CA-AKI) is multifactorial and involves several interrelated mechanisms, including inflammatory activation, apoptosis and necrosis, intracellular danger signaling pathways, shear stress-related tubular injury, renal vasoconstriction, medullary hypoxia, oxidative injury, and ischemic injury [7, 29–39]. Given the multifactorial pathophysiology of CA-AKI, we evaluated the renal expression of key inflammatory and apoptotic mediators, including TNF-α, IL-1β, IL-6, cytochrome c, and caspase-3, as well as the anti-apoptotic marker Bcl-XL. Although histopathological renal injury was reduced in the CNIB group compared with the untreated CN group, caspase-3 staining, Bcl-XL staining, and IL-6 expressionin renal tissue did not show statistically significant differences between groups. Interestingly, TNF-α, IL-1β, and Cyt-c expression levels were lower in the untreated CN group than in the CNIB group. These findings suggest that the molecular response to contrast injury and IB treatment may be more complex than expected and may not follow a classical anti-inflammatory or anti-apoptotic protective pathway.

Interestingly, caspase-3 immunostaining and IL-1β gene expression in the untreated CN group tended to be lower than in the sham group, although these differences did not reach statistical significance. In addition, cytochrome c expression was reduced in the untreated CN group despite the presence of markedly greater tubular injury and intracellular vacuolization on histopathological examination. These findings suggest that renal injury in this model may involve alternative mechanisms such as renal vasoconstriction, ischemia-related injury, oxidative stress, or contrast media–induced tubular toxicity rather than predominantly classical inflammatory or apoptotic activation.

To our knowledge, no previous experimental study has reported reduced caspase-3 or cytochrome c expression in CA-AKI models despite the presence of substantial histopathological renal injury. These findings may indicate that renal damage in this model is mediated, at least in part, through alternative mechanisms beyond classical inflammatory and apoptotic pathways, including oxidative stress, ischemia-related injury, vasoconstriction, tubular hypoxia, or damage-associated molecular pattern (DAMP)-related signaling pathways, including high mobility group box 1 protein (HMGB1). Accordingly, further in vivo and in vitro studies investigating these non-classical mechanisms are warranted to better characterize the complex pathophysiology of CA-AKI [29, 33, 40].

It should also be considered that the mechanisms underlying CA-AKI may vary according to the associated comorbidities and experimental conditions, including diabetes mellitus, chronic kidney disease, heart failure, renal hypoperfusion, and concomitant nephrotoxic exposure [1, 2]. In the present study, dimethyl sulfoxide (DMSO) was used as a solvent for IB and indomethacin because of their hydrophobic properties [19, 41–44]. Although vehicle administration was also performed in the sham group to minimize solvent-related bias, previous experimental studies have demonstrated that DMSO may exert biological effects on inflammatory and apoptotic signaling pathways [45–48]. Therefore, a potential modulatory effect of DMSO on baseline renal molecular markers cannot be completely excluded and should be considered when interpreting the apoptotic and inflammatory findings of the present study.

In the present study, IB was administered to evaluate its potential renoprotective effects in experimental CA-AKI. Previous studies have demonstrated that IB may exert context-dependent biological effects. At physiological or low concentrations, IB has been reported to exhibit antioxidant, anti-inflammatory, and anti-apoptotic properties in both in vivo and in vitro models [15, 17, 19, 49–54]. However, higher concentrations or prolonged exposure may promote cellular stress and tissue injury through activation of inflammatory and apoptotic signaling pathways, particularly in neuronal tissues [42, 55–58]. In addition, some experimental studies have suggested that bilirubin-associated cytotoxicity may not be entirely dose-dependent under certain cellular conditions [59–62]. These complex and potentially dual biological effects may partly contribute to the heterogeneous molecular findings observed in the present study.

In this study, CNIB group demonstrated reduced renal tubular vacuolization and histopathological injury compared to the untreated CN group, suggesting a structural protective effect of IB at the tissue level. In contrast, gene expression analysis revealed higher levels of IL-1β, TNF-α, and cytochrome c in the CNIB group compared to the untreated CN group. However, these molecular changes were not consistently reflected at the protein level, as no statistically significant differences were observed between the CNIB and sham groups in pro-inflammatory (IL-1β, TNF-α, IL-6) or pro-apoptotic markers (cytochrome c, caspase-3), based on both immunohistochemical and gene expression analyses. These findings may reflect dissociation between structural renal injury and classical inflammatory/apoptotic signaling in CA-AKI, potentially indicating the contribution of alternative mechanisms such as hemodynamic changes, oxidative stress, or microvascular dysfunction.

Additionally, caspase-3 expression was lower in both CN groups compared to the sham group, although this difference did not reach statistical significance. Similarly, Bcl-XL immunostaining showed a reduced trend in both treated and untreated CN groups compared to the sham group, without significant intergroup differences. Taken together, these findings suggest that IB may exert its renoprotective effects primarily at the structural level, while its influence on classical inflammatory and apoptotic markers in this model appears limited or not consistently reflected across molecular endpoints.

We observed a trend toward a pro-inflammatory response in the CNIB group; however, this did not reach statistical significance. Interestingly, pro-inflammatory cytokine expression (TNF-α, IL-1β) and cytochrome c levels were lower in the untreated CN group compared to the CNIB group, despite more pronounced histological tubular injury in the untreated group. This apparent dissociation between molecular markers and structural damage suggests that inflammatory and apoptotic signaling may not directly parallel the extent of histopathological injury in CA-AKI, and may reflect the involvement of additional non-classical pathways. This may indicate that renal structural injury in CA-AKI is driven by a combination of hemodynamic, oxidative, and microvascular factors beyond classical inflammatory and apoptotic pathways.

In this study, the small sample size in some analyses is a significant factor limiting the generalizability of the findings. Future studies with larger samples will enhance the reliability and validity of the results. The other limitations are as following, although gene expression and immunohistochemical analyses were performed, additional protein-level validation using methods such as ELISA or Western blot analysis could have provided stronger mechanistic support. Furthermore, other pathways involved in the pathophysiology of contrast-associated acute kidney injury, including vasoconstriction, renal ischemia, DAMPs, ROS, and additional intracellular signaling pathways, were not investigated in the present study. Electron microscopic evaluation could also have provided a more detailed assessment of ultrastructural renal injury.

Another limitation was the lack of direct glomerular filtration rate (GFR) measurement and additional renal functional biomarkers such as cystatin-C. Conventional GFR estimation formulas validated for humans are not directly applicable to experimental rat models. Accurate GFR assessment in rodents generally requires invasive or tracer-based techniques, including inulin or FITC-sinistrin clearance methods, which were not available within the technical infrastructure of the present study.

These findings raise important questions regarding the mechanisms underlying the renoprotective effects of IB in CA-AKI. In particular, the dissociation between structural protection and molecular inflammatory/apoptotic responses suggests that IB may act through pathways not fully captured by classical biomarkers. To better elucidate these mechanisms, further in vivo and in vitro studies are required, with a focus on alternative pathogenic pathways such as renal ischemia, vasoconstriction, ROS, and DAMPs. Additionally, key signaling cascades including mitogen activated protein kinases (MAPKs), NF-κB, c-Jun N-terminal kinase (JNK), and HMGB1, as well as biomechanical factors such as shear stress and contrast media - induced tubular injury, should be investigated. Together, these approaches may help clarify the complex and multifactorial nature of CA-AKI pathophysiology and the pleiotropic effects of IB.

## Conclusion

In summary, IB has been shown to exert anti-inflammatory, anti-apoptotic, and antioxidant effects in various tissues. To our knowledge, this is the first in vivo study to evaluate the therapeutic potential of exogenously administered IB in a CN model. Our findings suggest that IB attenuates renal tubular injury and vacuolization in experimental CA-AKI/CN. However, these structural protective effects were not consistently reflected in classical inflammatory and apoptotic markers, suggesting a more complex and potentially multifactorial mechanism of action. Further in vivo and in vitro studies are required to elucidate the precise molecular pathways underlying this renoprotective effect. Overall, these results suggest that IB may represent a potential candidate for further investigation in the prevention and treatment of CN/CA-AKI.

## Materials and methods

### Animals and experimental design

Twenty-four male Wistar albino rats (*Rattus norvegicus*) (8–10 weeks old, weighing 180–200 g) were obtained from the Experimental Medicine Research and Application Center of Karabük University, an authorized facility for animal research, where ethical approval was obtained. All experimental procedures were approved by the Animal Care Committee of Karabük University (protocol number: 2022/2/1). All methods were carried out in accordance with relevant guidelines and regulations. This study is reported in accordance with the ARRIVE guidelines. The animals were housed in standard cages at 25 °C under controlled humidity (50 ± 10%) with a 12-hour light/dark cycle and were provided with standard laboratory chow and water ad libitum. This study was conducted at the same center and included 24 male rats, which were randomly assigned into three groups (*n* = 8 per group): control (sham) group, contrast media (CN) group, and contrast media plus indirect bilirubin (CNIB) group. At the beginning of the experiment, all rats were weighed, and water was withheld for 24 h, while access to standard laboratory chow was maintained.

After the dehydration protocol, the rats were weighed again, and the CA-AKI induction protocol was applied to the CN and CNIB groups. Indomethacin (10 mg/kg; Cayman^®^, CAS No: 53-86-1, Ann Arbor, MI, USA) and L-NAME (10 mg/kg; Cayman^®^, CAS No: 51298-62-5, Ann Arbor, MI, USA) were administered i.p. to each rat in the CM and CMIB groups. Fifteen minutes later, iohexol (3 g/kg; Biemexol^®^ 350 mg/mL, Biem İlaç, Ankara, Turkey) was injected into the tail vein. Following the CA-AKI procedure, all rats were allowed free access to water.

At 12 and 24 h post-procedure, the CNIB group received i.p. injections of indirect bilirubin (30 mg/kg, Cayman^®^, CAS No: 635-65-4, Ann Arbor, MI, USA) dissolved in DMSO (CAS-Nummer: 67-68-5, Merck KGaA^®^, Darmstadt, Deutschland). The CN and sham groups received equivalent volumes of the vehicle (DMSO) via i.p. injection.

At 24 h following the CA-AKI procedure, all rats were placed in metabolic cages for 24 h to assess water intake and urine output. Forty-eight hours after CA-AKI induction, the Wistar albino rats were anesthetized by i.p. injection of ketamine (80–100 mg/kg, CAS Number: 1867-66-9, Cayman^®^, Ann Arbor, MI, USA) and xylazine (10–15 mg/kg, CAS Number: 7361-61-7, Merck KGaA^®^, St. Louis, Missouri, United States). Adequate depth of anesthesia was confirmed by the absence of reflex responses prior to euthanasia. Animals were then euthanized by cervical dislocation in accordance with institutional and international ethical guidelines. Terminal blood samples were collected from the abdominal aorta for the measurement of BUN and Scr. Due to technical sample loss and incomplete urine collection during metabolic cage measurements, the sample size differed slightly between some analyses. Twenty-four-hour urine samples were collected to determine urinary creatinine and urea levels. Both kidneys were harvested for histological and immunohistochemical evaluation, as well as for gene expression analysis.

### Biochemical analysis of urine and serum

Serum was obtained by centrifuging the collected blood samples at 3000 g for 10 min. Serum and urine levels of creatinine and urea were analyzed spectrophotometrically using the Cobas c311 analyzer (Roche Diagnostics^®^ GmbH, Mannheim, Germany).

### Kidney histology

The left kidneys were excised, fixed in 10% formalin for 24 h, embedded in paraffin, sectioned at 5 μm thickness, and stained with hematoxylin and eosin (H&E). Morphological changes in renal tissue were evaluated under a light microscope (Leica DM4000; Leica Instruments^®^, Wetzlar, Germany) at ×200 magnification. Tubular cell degeneration, vacuolization, and necrotic changes were assessed semi-quantitatively using a scoring system ranging from 0 to 4 as follows: 0 = no injury; 1 = mild (0% − 25%); 2 = moderate (25% − 50%); 3 = severe (50% − 75%); and 4 = very severe (75% − 100%). The histopathological evaluations of the kidneys were performed by a single experienced veterinary pathologist who was blinded to the experimental groups.

### Immunohistology

#### Caspase-3 and Bcl-XL immunohistochemical (IHC) staining and evaluation

Immunohistochemistry (IHC) is a method used to identify highly specific proteins in tissue sections. In this study, IHC was performed on formalin-fixed, paraffin-embedded kidney tissue samples.

Tissues were fixed in 10% neutral buffered formalin for 24 h, then processed using a Leica ASP300S Fully Enclosed Vacuum Tissue Processor (Leica Instruments^®^, Wetzlar, Germany). The samples were embedded using the Leica EG1150 C Cold Plate for Modular Tissue Embedding. Tissue sections were cut at a thickness of 3 μm from both contrast nephropathy and healthy renal paraffin blocks using a Leica RM2125RT Rotary Microtome (Leica Instruments^®^, Wetzlar, Germany).

Following sectioning, slides were subjected to automated IHC staining using the BOND-MAX Fully Automated IHC and ISH Staining System (Leica Instruments^®^, Nußloch, Germany). The protocol included baking, deparaffinization, and incubation steps. Antigen retrieval was performed using heat-induced epitope retrieval (HIER) with EDTA buffer (pH 9.0) for 20 min. Endogenous peroxidase activity was blocked using hydrogen peroxide for 10 min.

For primary antibody incubation:

- **Caspase-3 antibody** (Abcam, ab4051, rabbit polyclonal, Lot No: Gr3265151-5; 152 Grove Street, Third Avenue, Waltham, MA, USA 02453) was applied at a 1:100 dilution and incubated for 30 min.
- **Bcl-XL antibody** (St John’s Laboratory, London, UK, E16 2RD) was applied at a 1:50 dilution and incubated for 20 min.

Detection was performed using the Leica HRP-conjugated polymer detection kit (DS9800, New Castle, United Kingdom). The steps included post-polymer incubation for 12 min, polymer incubation for 12 min, DAB chromogen for 10 min, and hematoxylin counterstaining for 10 min. Slides were washed with washing solution between each step, followed by dehydration and mounting with Entellan.

Scoring of Caspase-3 and Bcl-XL Staining:

The immunoreactivity of Caspase-3 and Bcl-XL was evaluated by assessing the percentage of positively stained cells in contrast nephropathy and healthy renal tissue samples. The staining intensity was classified into three categories: Score 1 (Negative): 0–25% positive staining, Score 2 (Moderate): 25–75% positive staining and Score 3 (Strong Positive): >75% positive staining.

Histopathological and immunohistochemical evaluations were performed in a blinded manner, with all slides coded as A, B, and C and the pathologist unaware of group allocation during scoring.

### Real time – PCR/Gene expression

The right kidneys were used for gene expression analysis. RNA was isolated from renal tissue using the High Pure RNA Isolation Kit (Cat No: 11 828 665 001, Roche, USA), and complementary DNA (cDNA) was synthesized using the OneScript^®^ Plus cDNA Synthesis Kit (Cat NO: G236, ABM, Canada).

Gene expression levels of TNF-α, IL-6, IL-1β, cytochrome c, and β-actin were analyzed using specific primers and BlasTaq™ 2X qPCR MasterMix (Cat NO: G891, ABM, Richmond. BC, CANADA) on an BioRAD CFX96 Real-Time PCR system (BioRAD, California, USA). Cycle threshold (Ct) values were recorded, and relative gene expression levels of TNF-α, IL-6, IL-1β, and cytochrome c were calculated using the 2^–ΔΔCt^ method, with β-actin serving as the housekeeping gene.

### Statistical analysis

Since the number of subjects in each group was below 30, the Shapiro-Wilk test was used to assess data normality. For normally distributed (parametric) data, one-way ANOVA followed by the Least Significant Difference (LSD) post-hoc test was used for group comparisons. For non-normally distributed (non-parametric) data, the Kruskal-Wallis test was used for overall group comparisons. Pairwise comparisons for histopathologic and immunohistochemical analyses were performed using Bonferroni-adjusted Mann-Whitney U tests. Accordingly, the significance threshold was adjusted from *p* < 0.05 to *p* < 0.017 for multiple comparisons. For other non-parametric variables, pairwise comparisons were performed using the Mann-Whitney U test. Statistical analyses were performed using SPSS software, and graphs were generated using GraphPad Prism version 5.01 (GraphPad Software^®^, La Jolla, California, USA).

## Data Availability

The data supporting the findings of this study are included within the article.
